# Design and Synthesis of Copper Nanobiomaterials with Antimicrobial Properties

**DOI:** 10.1021/acsbiomedchemau.2c00089

**Published:** 2023-04-11

**Authors:** Clara Ortega-Nieto, Noelia Losada-Garcia, Benevides C. Pessela, Pilar Domingo-Calap, Jose M. Palomo

**Affiliations:** † Instituto de Catalisis y Petroleoquimica (ICP), CSIC, Marie Curie 2, 28049 Madrid, Spain; ‡ Institute of Food Science Research (CIAL, CSIC-UAM), Nicolás Cabrera, 9, Cantoblanco, 28049 Madrid, Spain; § Institute for Integrative Systems Biology (I^2^SysBio), Universitat de València-CSIC, 46980 Paterna, Spain

**Keywords:** copper, nanoparticles, nanostructured materials, metal bionanohybrids, antimicrobial activity

## Abstract

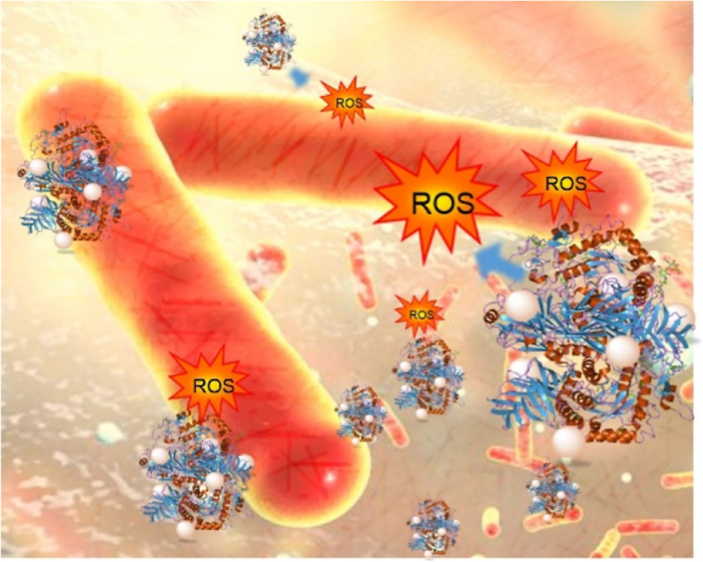

In this work, nanostructured copper materials have been designed, synthetized, and evaluated in order to produce a more efficient and sustainable copper bionanohybrid with catalytical and antimicrobial properties. Thus, conditions are sought where the most critical steps are reduced or minimized, such as the use of reducing agents or the cryogenization step. In addition, the new materials have been characterized through different techniques, and their oxidative and reductive capacities, as well as their antimicrobial activity, have been evaluated. The addition of different quantities of a reducing agent in the synthesis method generated copper bionanohybrids with different metallic species, nanoparticles sizes, and structures. The antimicrobial properties of the bionanohybrids were studied against different strains of Gram-positive and Gram-negative bacteria through two different methods: by counting the CFU and via the disk diffusion test, respectively. The bionanohybrids have demonstrated that different efficiencies depending on the bacterial strain were confronted with. The **Cu-PHOS-100% R** hybrids with the highest percentage of reduction showed the best antimicrobial efficiency against *Escherichia coli* and *Klebsiella pneumoniae* bacteria (>96 or >77% in 4 h, respectively) compared to 31% bacteria reduction using **Cu-PHOS-0% R**. Also, the antimicrobial activity against *Bacillus subtilis* materials was obtained with **Cu-PHOS-100% R** (31 mm inhibition zone and 125 μg/mL minimum inhibitory concentration value). Interestingly, the better antimicrobial activity of the nanobiohybrids against Gram-positive bacteria *Mycobacterium smegmatis* was obtained with some with a lower reduction step in the synthesis, **Cu-PHOS-10% R** or **Cu-PHOS-20% R** (>94% bacterial reduction in 4 h).

## 1 Introduction

In last decades, the emergence of antibiotic-resistant bacteria and its related consequences has raised the interest for the development of new antimicrobial materials. Historically, the antimicrobial capacity of different metals, such as Ag, Au, Cu, Zn, or Ti, has been known, studied, and used for a variety of applications.^1,2^ However, in recent years, the use of those metals as nanoparticles (NPs) or nanostructures has been demonstrated to be more efficient as bulk materials due to their high surface-to-volume ratio. In addition, NPs develop their antimicrobial action through different mechanisms compared to the traditional antimicrobial agents,^3,4^ which is an advantage against the resistant bacteria.

In particular, copper is a metal which stands out for its antimicrobial capability, abundance, and competitive price compared to others, such as Au or Ag. Thus, copper NPs (CuNPs) have been the subject of studies, and their application has been explored in various fields, such as in catalysis and bioremediation or as antimicrobial agents.^5,6^ Nevertheless, CuNPs have as disadvantages their low stability under atmospheric conditions, which could lead to an undesired oxidation, high cost, and low sustainability of the methods that generate them in a pure form. Therefore, several methods have been developed based on the stabilization of CuNPs through more complex structures, such as metabolites, proteins, biopolymers, or even plant extracts.^7−14^

**Figure 1:**
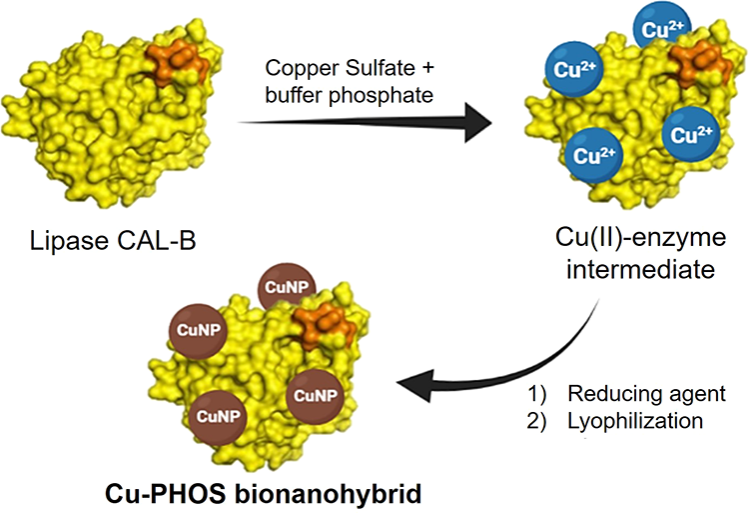
Schematic diagram illustrating the synthesis route of copper bionanohybrids.

However, the use of isolated proteins or enzymes as a scaffold present important advantages: special conditions for metal coordination that support biomineralization processes, allow control over particle size and growth, and avoid aggregation of generated NPs (Figure 1). They stabilize the NPs, produce monodisperse NPs, control the shape and size of NPs, and allow to synthetize them in water at room temperature in a more sustainable and “green” way.^15−24^ The three-dimensional structure of the enzyme acts as a scaffold of the in situ generated NPs for obtaining the final heterogeneous material. This methodology differs clearly from the many examples of immobilization of enzymes in metal NPs.^25^

The three steps in the hypothesized mechanism for creating these enzyme–metal NP biohybrids are (i) ionic interaction of metal (Me) ions with various protein amino acid residues; (ii) nucleation, which is the reduction of Me^*n*+^ to Me(0); and (iii) coalescence, which is the growth of Me NPs (Figure S1).

Understanding the mechanism requires taking into account how amino acids and small protein peptide sequences contribute to the synthesis of the entire nanobiohybrid.

The enzyme’s peptide sequences are quickly coated with soluble Me^*n*+^ ions in the first step (intermediate binding affinity), which reduces the enzyme’s solubility and acts as a cross-linker between the molecules of the enzyme (initial fast precipitation). In the case of copper, potential redox makes necessary the addition of an external reducing agent to obtain Cu(i) species from Cu(ii) or even Cu(0).

Finally, the deposition of the continuously formed metal atoms on the previously generated metal nuclei surface leads to the NP growth, generating the final metal NPs.

At this point, the protein structure also regulates the final NP size formation, affecting to the final oxidative species. The presence of the protein as a matrix makes a microstructure containing small NPs as global being therefore different mechanisms as simple-free NPs.

Here, different copper bionanohybrids were synthesized using lipase B from *Candida antarctica* (CAL-B) as an enzyme scaffold.

This lipase was selected as a scaffold because it is an available commercial enzyme, highly pure sample, monomeric structure, with high stability in a broad pH range (from 3 to 11) and temperatures (4 to 50 °C) or in the presence of a co-solvent (5–40%).

**Figure 2:**
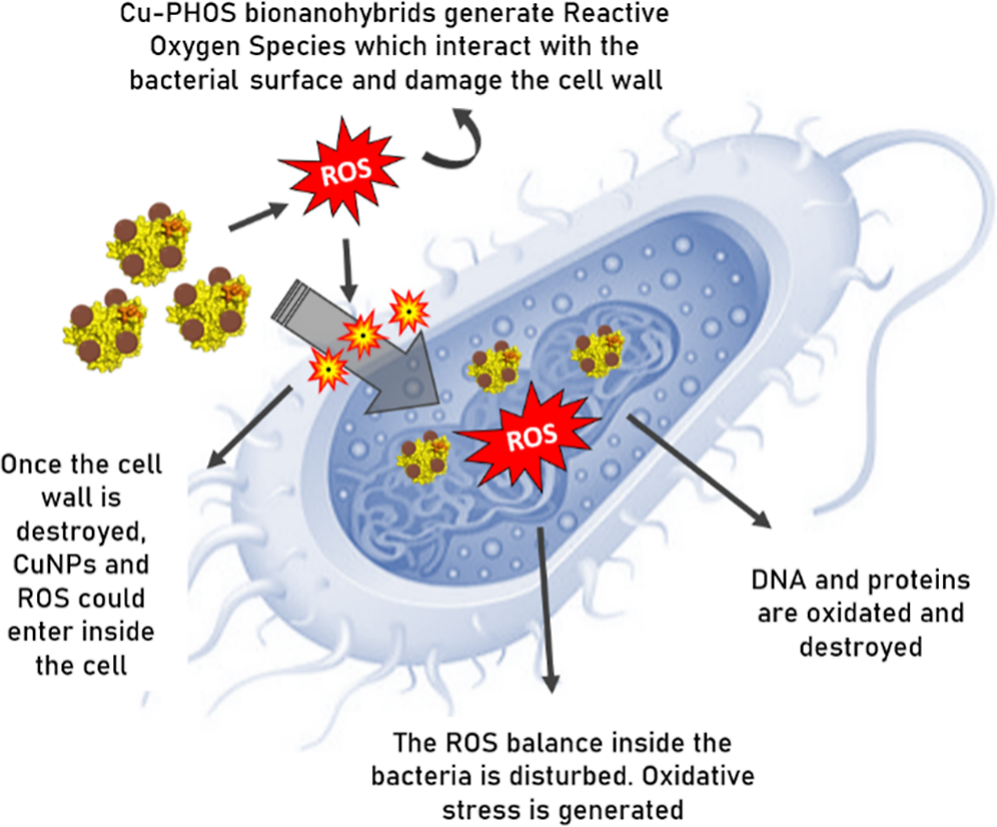
Antibacterial proposed mechanism of Cu nanobiomaterials.

Regarding to the action mechanisms of CuNPs against bacteria, they have not yet been clearly described. However, most authors agree on the following points: CuNPs produce reactive oxygen species (ROS), which interacts with the cell wall until it is destabilized and NPs are able to pass through it. Even, cellular leakage may occur. In the case of bionanohybrids, CuNPs do not agglomerate on the wall surface as free CuNPs do, which is a disadvantage as discussed previously. Afterward, NPs can disturb the ROS balance inside the bacteria, which generates oxidative stress. Also, they are capable of inhibiting the synthesis of DNA and proteins (Figure 2).^3,26^

On the other hand, previous studies have shown that by the use of other copper bionanohybrids similar to those of the present work, oxidation–reduction reactions take place through radical mechanisms.^8−10^ In any case, further studies would be necessary to understand better the mechanism of ROS generation, the intermediate species involved (such as ^•^OH or O_2_^•–^ radicals), and the destabilization of the cell wall structure.

Therefore, one of the interesting elements in the preparation of new copper nanomaterials with improved antimicrobial efficiency would be the study of the effect of the reducing step.

In a first approach to evaluate the antimicrobial capacity of new bionanohybrids, the use of model reactions can be useful to get an idea of their behavior before performing antimicrobial assays. In this way, model reactions based on oxidation of *p*-aminophenol (pAP) and reduction of *p*-nitrophenol (pNP), which have previously been studied in the laboratory, can be used for such purposes.^9,15,16,22^ It should be stressed that the ROS mechanism, based on radical generation, can be emulated through the Fenton oxidation reaction of pAP.

Therefore, the aim of this work is to design new copper bionanohybrids to produce more efficient and sustainable materials with antimicrobial properties. For this purpose, in this work, different parameters in the synthesis of the bionanohybrids using different quantities of a reducing agent have been evaluated.

## 2 Materials and Methods

### 2.1 Materials

Lipase B from *C. antarctica* (CAL-B) solution was supplied by Novozymes (Copenhagen, Denmark). Copper(II) sulfate pentahydrate [Cu_2_SO_4_·5H_2_O], hydrogen peroxide (33%, v/v), sodium hydrogen carbonate, sodium phosphate, sodium borohydride, and pNP were purchased from Sigma-Aldrich (St. Louis, MO, USA). pAP was from Alfa Aesar (Ward Hill, MA, USA).

### 2.2 Characterization and Analysis Techniques

X-ray diffraction (XRD) patterns were obtained using a Texture Analysis D8 ADVANCE Diffractometer (Bruker, Billerica, MA, USA) with Cu Kα radiation. Scanning electron microscopy (SEM) imaging was performed on a TM-1000 microscope (Hitachi, Tokyo, Japan). Transmission electron microscopy (TEM) and high-resolution TEM microscopy (HR-TEM) images were obtained on a 2100F microscope (JEOL, Tokyo, Japan) equipped with an EDX detector INCA x-sight (Oxford Instruments, Abingdon, UK). Interplanar spacing in the nanostructures was calculated by using the inversed Fourier transform (FT) with the GATAN digital micrograph program (Corporate Headquarters, Pleasanton, CA, USA). Spectrophotometric analyses were run on a V-730 spectrophotometer (JASCO, Tokyo, Japan). Inductively coupled plasma–optical emission spectrometry (ICP–OES) was performed on an OPTIMA 2100 DV instrument (PerkinElmer, Waltham, MA, USA). To recover the bionanohybrids, a Biocen 22 R (Orto-Alresa, Ajalvir, Spain) refrigerated centrifuge was used. Chromatographic analyses were run at 25 °C using a high-performance liquid chromatography (HPLC) pump PU-4180 (JASCO, Tokyo, Japan) and a UV-4075 UV–vis detector (JASCO, Tokyo, Japan).

### 2.3 Synthesis of Cu-PHOS Bionanohybrids

3.6 mL of CAL-B solution (10.34 mg/mL, determined by Bradford assay)^27^ was added to 60 mL of buffer 0.1 M (sodium phosphate pH 7) in a 250 mL glass bottle containing a small magnetic bar stirrer. Then, 600 mg of Cu_2_SO_4_·5H_2_O (10 mg/mL) was added to the protein solution, and it was stirred for 1 h at room temperature. After 1 h, 6 mL of NaBH_4_ (300 mg) aqueous solution (1.2 M) was added to the mixture (in 2 times of 3 mL). The solution turned rapidly black, and it was stirred for 30 min. After the reduction step, the mixture was centrifuged at 8000 rpm for 5 min (10 mL per Falcon-type tube). The generated pellet was resuspended in 15 mL of water. It was centrifuged again at 8000 rpm for 5 min, and the supernatant was removed. The process was repeated twice more. Finally, the supernatant was removed, and the pellet of each Falcon was resuspended in 2 mL of water. Each solution was collected in a cryotube, frozen with liquid nitrogen, and lyophilized for 16 h. After that, 0.20 g of a black solid was obtained. The bionanohybrid was called **Cu-PHOS-100% R** (Figure S2).

Other hybrids were synthetized reducing the amount of NaBH_4_ by up to 50, 30, 20, 10, and 0%. The different hybrids were called **Cu-PHOS-50% R**, **Cu-PHOS-30% R**, **Cu-PHOS-20% R**, **Cu-PHOS-10% R**, and **Cu-PHOS-0% R**. The amounts of the different hybrids obtained after the synthesis were from 0.23 to 0.34 g. The color of the hybrids lightened from black to light blue as the amount of NaBH_4_ was reduced.

### 2.4 Synthesis of Cu-BIC Bionanohybrids

The same synthesis method as for **Cu-PHOS-100% R** bionanohybrid was performed but changing the phosphate buffer (0.1 M, pH 7) to bicarbonate buffer (0.1 M, pH 10), obtaining a new one called **Cu-BIC-100% R**. An amount of 0.12 g of brownish black powder was weighed after the lyophilization.

### 2.5 Evaluation of Metallic Activity of Different Bionanohybrids in the Reduction of pNP to pAP Reaction

Cu-PHOS bionanohybrids were used as catalysts in the transformation of pNP to pAP. First, a solution of pNP 5 mM was prepared in distilled water. Then, 15 mg of NaBH_4_ was added to 2 mL of pNP solution and stirred for 30 s to form the phenolate ion (bright yellow) at room temperature. After that, 3 mg of the catalyst was added to the solution. The reaction was followed by measuring the transformation of pNP to pAP via a spectrophotometer in the range from 290 to 600 nm. Samples were centrifuged and diluted in plastic cuvettes of 1 cm path length with a 1:100 dilution. They were taken at different times until a total pNP transformation to pAP, keeping the reaction stirred on an orbital shaker. Experiments were performed in duplicate or triplicate.

### 2.6 Evaluation of Metallic Activity of Hybrids in the Fenton Oxidation of pAP Reaction

Cu-PHOS bionanohybrids were used as catalysts in the degradation of pAP. First, a solution of pAP (100 ppm) was prepared in distilled water, and 0.5% (v/v) H_2_O_2_ was added. To start the reaction, 3 mg of the catalyst was added to 10 mL of pAP solution and stirred gently at room temperature with a small magnetic bar stirrer. Samples of 150 μL were taken at different times and centrifuged. Then, 100 μL of the supernatant was diluted 1:10 (with an elution solvent mixture) and injected in HPLC. The elution solvent was a mixture of water–acetonitrile in an 85:15 ratio. A Kromasil C-8 column (5 μm, 150 mm × 4.6 mm) was used, and the injection volume was 10 μL. The degradation of pAP was followed with an UV–vis detector at 254 nm and a flow rate of 0.7 mL/min. Under these conditions, the retention times of pAP and H_2_O_2_ were 2.37 and 4.30 min, respectively.

### 2.7 Antimicrobial Assay: Disk Diffusion Method

The antimicrobial activity was determined using the disk diffusion method. The strain used was *Bacillus subtilis* 365 from CECT collection. For this purpose, 100 μL of the strain was inoculated in 10 mL of Luria–Bertani (LB) liquid media broth. The bacteria were grown overnight at 37 °C on an orbital shaker at 150 rpm. The absorbance was measured at 600 nm, obtaining the optical density value of 2. Then, 50 μL of bacterial broth was spread on a plate of solid LB medium (53 mm of diameter). After that, a disk (9 mm of diameter) impregnated with a suspension of the bionanohybrid of interest was placed in each plate. Different concentrations (1250, 625, 500, 250, and 125 ppm) were tested in order to obtain the minimum inhibitory concentration (MIC). The plates were incubated upside down at 37 °C. The diameter of the inhibition zone was measured after 48 h, including the diameter of the disk. Controls were performed with water and CAL-B lipase without interfering with bacterial growth. A positive control was initially made with ampicillin. All experiments were performed in duplicate.

### 2.8 Antimicrobial Assay: Colony-Forming Units

The bacterial viability in the presence of the bionanohybrid was tested against different bacterial models. The bacteria used for this experiment were *Escherichia coli* C IJ1862 obtained from Prof. James J. Bull, *Klebsiella pneumoniae* from the Serum Statens Institute Collection, and *Mycobacterium smegmatis* mc^2^155 from the ATCC collection. First, 2.5 mg of each nanohybrid was added to 2 mL of Milli-Q water, and the mixture was stirred well, getting a final concentration of 1250 ppm. Then, 50 μL of the previous preparation was added to 400 μL of the LB broth supplemented with 1 mM CaCl_2_. After that, 50 μL of a stationary culture of bacteria was inoculated into the mixture to a final concentration of ∼2 × 10^9^ CFU/mL for *E. coli* and *K. pneumoniae*, and ∼2 × 10^7^ CFU/mL for *M. smegmatis*, and incubated at 37 °C at 900 rpm for 4 h. After the exposure time, the samples were serially diluted and cultured on LB agar plates, and final bacterial concentrations (CFU/mL) were determined by counting the colonies. Controls were done in parallel in the absence of the bionanohybrid or with lipase to determine the bacterial death over time (Figure S3). All experiments were performed in triplicate.

The following equations were applied to obtain the bacterial concentration and the percentage reduction in bacterial concentration
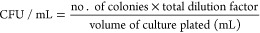

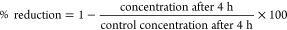


## 3 Results and Discussion

### 3.1 Synthesis and Characterization of Cu-PHOS Bionanohybrids

The synthesis of CuNPs biohybrids has been performed following the mild conditions method described in detail in the Materials and Methods section. Cu-PHOS hybrids were synthetized in water, without solvents at room temperature by the combination of an aqueous solution of lipase B from *C. antarctica*, copper sulfate, sodium phosphate 0.1 M at pH 7, and sodium borohydride as the reducing agent. After that, they were washed and lyophilized following the steps described in the Materials and Methods section.

Based on this method, different conditions were evaluated in order to produce a more efficient and sustainable bionanohybrid with catalytical and antimicrobial properties.

**Figure 3:**
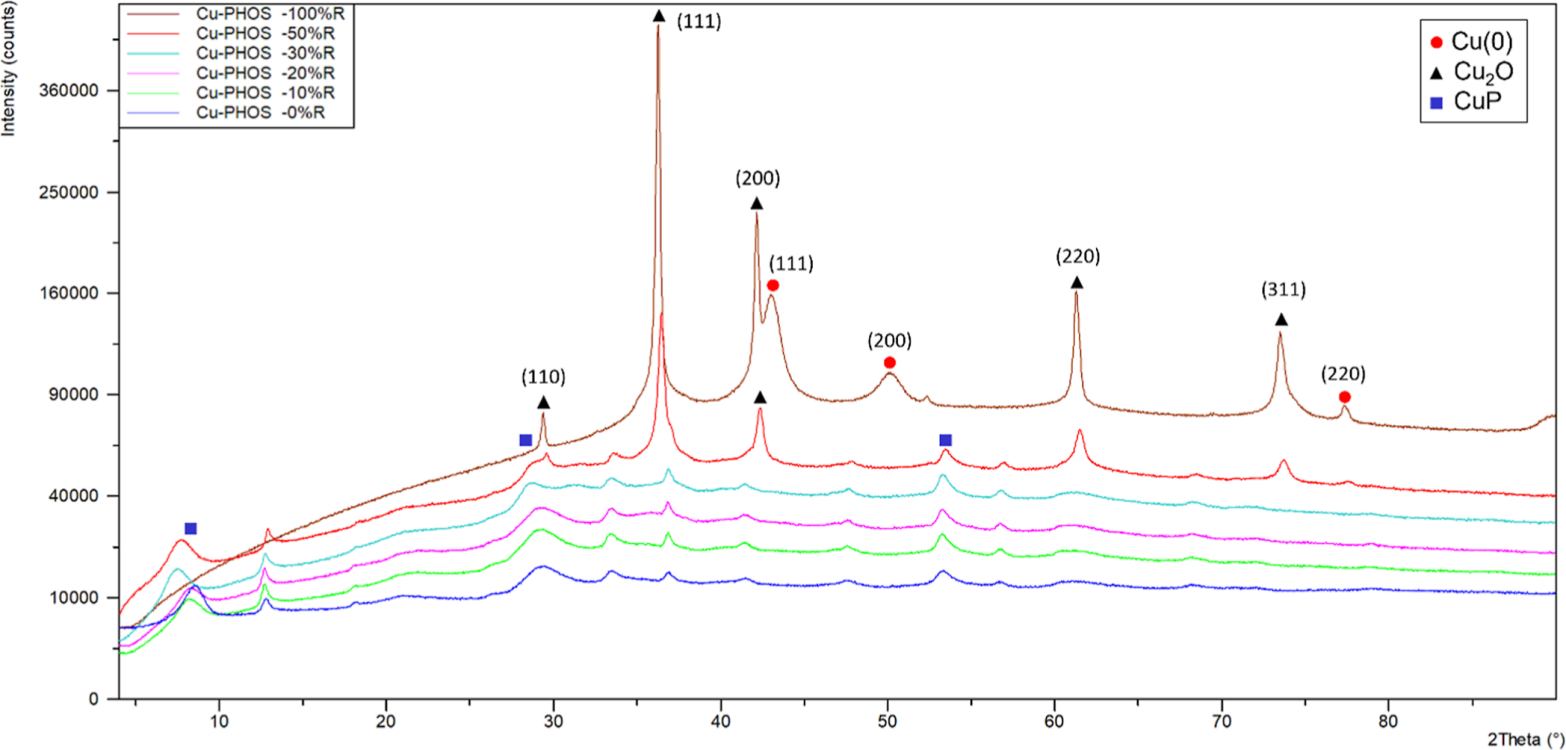
XRD spectrum of Cu-PHOS nanohybrids. CuP = Cu_3_(PO_4_)_2_.

First, the bionanohybrid called **Cu-PHOS-100% R** was synthesized, following the steps described in the Materials and Methods section. XRD analysis was performed (Figure 3), revealing the presence of two species, Cu(I) in the form of Cu_2_O and Cu(0). The diffraction peaks of (110), (111), (200), (220), and (311) from the XRD pattern matched well with the Cu_2_O standard data (JCPDS card no. 05-0667). The (111), (200), and (220) peaks matched well with the Cu(0) standard (JCPDS card no. 04-0836). Also, peaks matched well with the Cu_3_(PO_4_)_2_ standard (JCPDS card no. 00-022-0548).

After that, other molecules with less reducing power were evaluated (ascorbic acid and cysteine), but the results were not satisfactory, and these bionanohybrids were discarded (data not shown).

Then, the amount of sodium borohydride in the synthetic protocol was performed, from 50 to 0%, obtaining the bionanohybrids called **Cu-PHOS-50% R**, **Cu-PHOS-30% R**, **Cu-PHOS-20% R**, **Cu-PHOS-10% R**, and **Cu-PHOS-0% R**, respectively. The bionanohybrids obtained were analyzed by XRD (Figure 3), revealing different species according to the reduction degree. **Cu-PHOS-50% R** showed the presence of Cu_2_O and, compared to **Cu-PHOS-100% R**, Cu(0) has disappeared in its XRD pattern. On the other hand, it can be appreciated that in **Cu-PHOS-50% R**, other copper species begin to appear, which correspond with Cu_3_(PO_4_)_2_ (matched well with JCPDS card no. 22-0548).^10^ In **Cu-PHOS-30% R**, **Cu-PHOS-20% R**, **Cu-PHOS-10% R**, and **Cu-PHOS-0% R**, this conversion to Cu_3_(PO_4_)_2_ species was complete and nothing remains from Cu(0) or Cu_2_O species.

Thus, it was observed that as the amount of borohydride was reduced, the copper phosphate species appeared, while the most oxidized copper species disappeared, until it reached a limit in which there was no significance differences between the XRD patterns of less reduced bionanohybrids.

**Figure 4:**
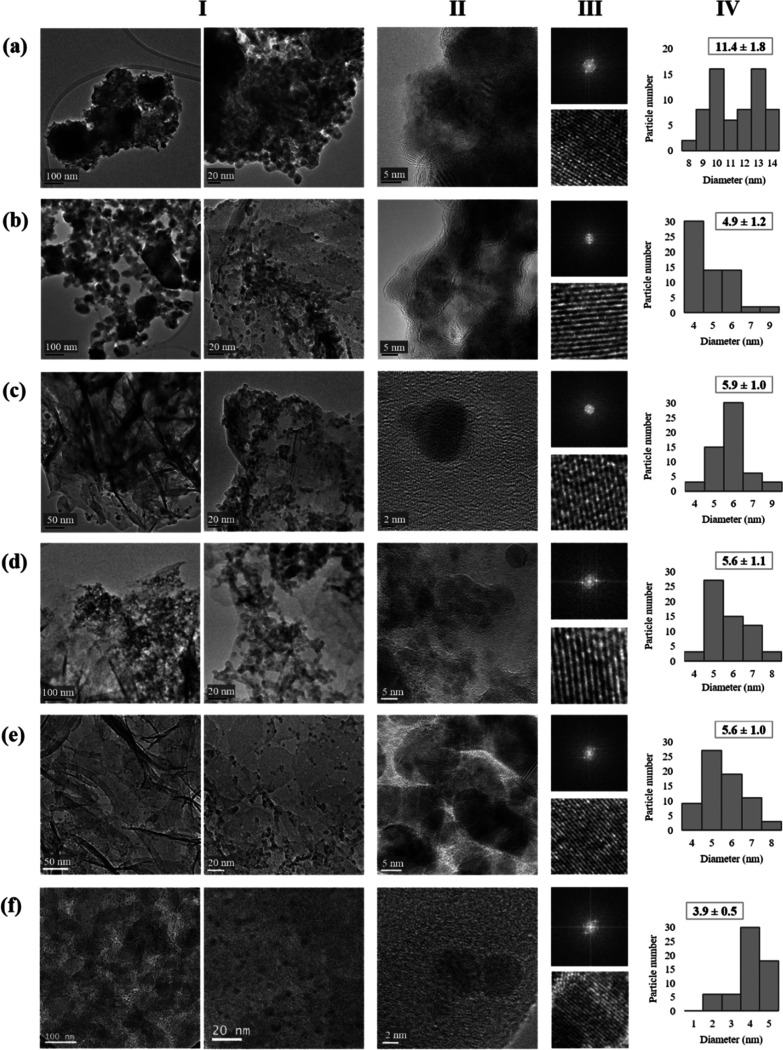
Characterization of Cu-PHOS bionanohybrids: (a) **Cu-PHOS-100% R**, (b) **Cu-PHOS-50% R**, (c) **Cu-PHOS-30% R**, (d) **Cu-PHOS-20% R**, (e) **Cu-PHOS-10% R**, and (f) **Cu-PHOS-0% R**. (I) TEM images; (II) HR-TEM images; (III) FT and inverse FT; and (IV) NP size distribution.

TEM analyses demonstrated that the methodology designed for the synthesis of bionanohybrids was suitable for synthetizing small crystalline NPs. Different average diameter particle sizes were found depending on the synthesis method, in a range between 4 and 11 nm, as is shown in Figure 4.

The highest NP size was found in **Cu-PHOS-100% R**, in which 10 and 13 nm predominated and the average NP size was 11.4 nm (Figure 4a). As the amount of NaBH_4_ in the nanohybrids was reduced, the NP sizes also decreased. An average particle size of 4.9, 5.9, 5.6, 5.6, and 3.9 nm was found for **Cu-PHOS-50% R**, **Cu-PHOS-30% R**, **Cu-PHOS-20% R**, **Cu-PHOS-10% R**, and **Cu-PHOS-0% R** bionanohybrids, respectively (Figure 4b–f).

In addition, nanostructures with different morphologies can be seen in TEM images. In **Cu-PHOS-10% R**, the formation of nanostructures was observed with a size of approx. 4 nm in width and 50–260 nm in length (Figure 4e). These structures were also found in **Cu-PHOS-20% R** and **Cu-PHOS-30% R** but with increasing width (Figure 4c,d). In **Cu-PHOS-50% R**, the particles aggregate into larger structures, from 30 nm to more than 250 nm (Figure 4b).

These bigger structures can also be seen in **Cu-PHOS-100% R**, where the particle aggregation was maximal. In contrast, in **Cu-PHOS-0% R**, unique spherical NPs were found (Figure 4f).

This seems to indicate that an increase of the reducing agent in bionanohybrid synthesis generates larger and agglomerated nanostructures. Similar observations have been reported previously by changing the oxidation state of the NPs.^18,28^ On the other hand, the decrease in particle size corresponds with a decrease in the percentage of the reducing agent, except in the **Cu-PHOS-50% R** hybrid, whose average particle size is smaller than **Cu-PHOS-30% R**, **Cu-PHOS-20% R**, and **Cu-PHOS-10% R** and higher than **Cu-PHOS-0% R**. This could be because in **Cu-PHOS-50% R**, the great aggregation of NPs may have hindered the counting of particles larger than 7 nm.

The copper content of the final bionanohybrids was determined by ICP–OES analyses. The percentages of copper in the different bionanohybrids were 45, 37, 55, 29, 27, and 32% for **Cu-PHOS-100% R**, **Cu-PHOS-50% R**, **Cu-PHOS-30% R**, **Cu-PHOS-20% R**, **Cu-PHOS-10% R**, and **Cu-PHOS-0% R**, respectively.

In other variation of the protocol, the lyophilization was removed as a critical step in the possible scaling up of the process. All the Cu-PHOS bionanohybrids with different percentages of NaBH_4_ were synthetized following the same procedure as before but removing the cryogenic freezing and the lyophilization step and keeping them in a water suspension after the washing. However, the non-lyophilized bionanohybrids were not as stable as the lyophilized ones after a few weeks, neither so efficient in the model reactions evaluated, so they were discarded (data not shown).

Furthermore, the use of carbonate buffer rather than phosphate buffer was tested generating a Cu-BIC bionanohybrid. As previous studies have demonstrated before, the predominant specie in **Cu-BIC-100% R** was Cu(0), with a slight content of Cu_2_O, and TEM analysis revealed the formation of very crystalline NPs with a size of 6 nm in diameter.^12^

### 3.2 Evaluation of Metallic Activity of Copper Bionanohybrids

As previously discussed, pAP oxidation and pNP reduction are model reactions which have been used before and evaluated in the group in order to assess the metallic behavior of new bionanohybrids designed and synthetized.

**Figure 5:**
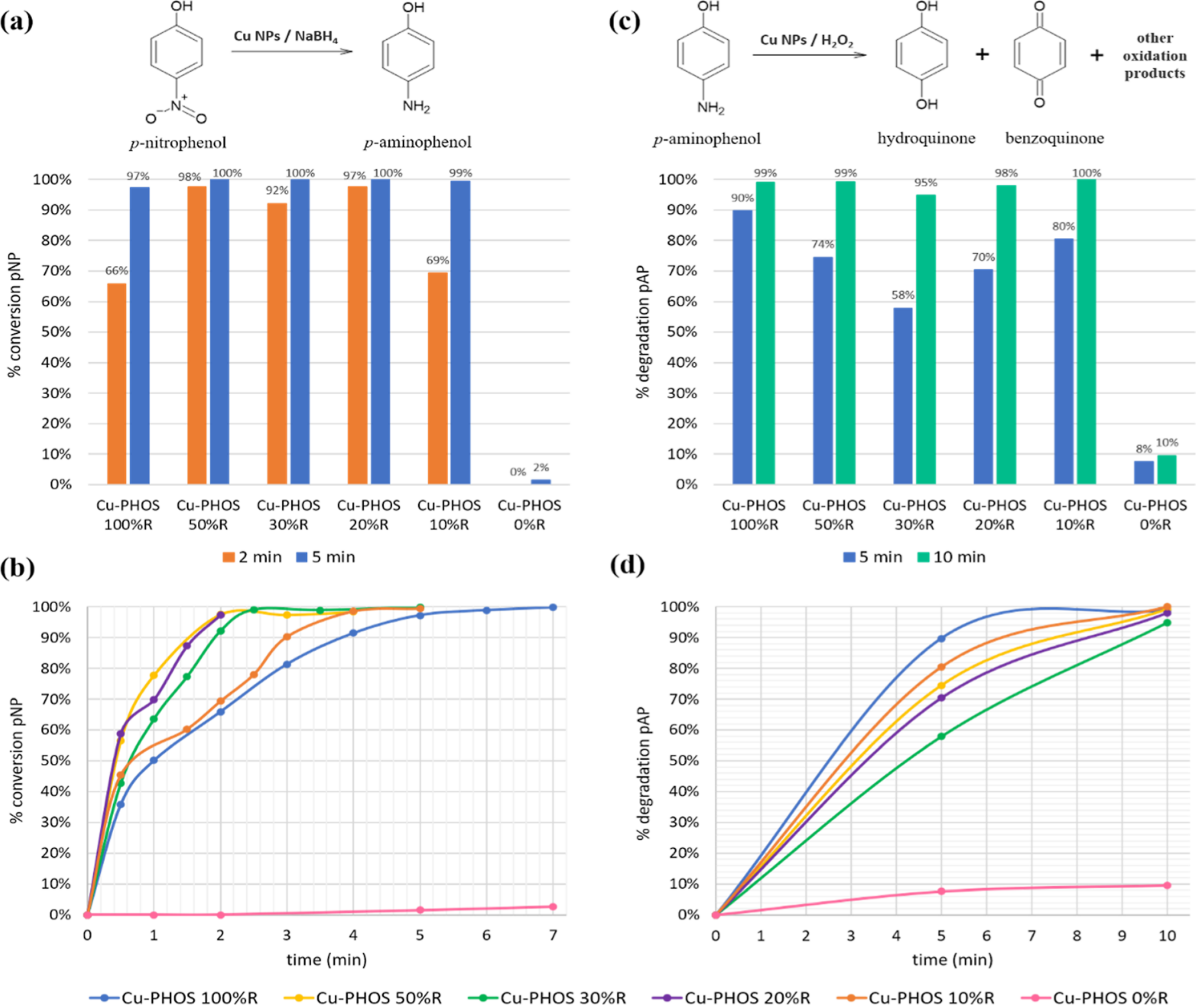
(a) pNP (5 mM) activity of Cu-PHOS hybrids at 2 min (orange) and 5 min (blue); (b) profile of bionanohybrid activity in pNP (5 mM) reaction; (c) pAP (100 ppm) activity of Cu-PHOS hybrids at 5 min (blue) and 10 min (green); and (d) profile of bionanohybrid activity in pAP (100 ppm) reaction.

Cu-PHOS bionanohybrids were used as catalysts in the transformation of pNP (5 mM) to pAP (Figure 5a,b). Bionanohybrid (3 mg) was added to the pNP solution with 15 mg of NaBH_4_. Under these conditions, the total conversion of pNP had practically been reached in 5 min with all the bionanohybrids except **Cu-PHOS-0% R**, which achieved only a 2%. In 2 min, more than 90% of pNP was transformed with **Cu-PHOS-50% R**, **Cu-PHOS-30% R**, and **Cu-PHOS-20% R** and more than 65% with **Cu-PHOS-100% R** and **Cu-PHOS-10% R**. These results highlight the great reductive capacity that the bionanohybrids with a percentage of reducing agent have, even if it is small, compared with the non-reduced one.

Cu-PHOS biohybrids were also tested as a catalyst in pAP (100 ppm) degradation (Figure 5c,d). The catalyst (3 mg) was added to the pAP solution with 0.5% (v/v) H_2_O_2_. Under these conditions, more than 95% of pAP was degraded in 10 min with all the bionanohybrids except **Cu-PHOS-0% R**, which only achieved a 10%.

In 5 min, **Cu-PHOS-100% R** got a 90% degradation of pAP, being the highest percentage at that time over all the catalysts. 80, 74, 70, 58, and 8% Are the pAP degradation percentages for **Cu-PHOS-10% R**, **Cu-PHOS-50% R**, **Cu-PHOS-20% R**, **Cu-PHOS-30% R**, and **Cu-PHOS-0% R**, respectively. As occurs in pNP reaction, the results were better for all the reduced hybrids compared with the non-reduced ones, although in this case, the oxidative capacity was evaluated. This indicates that the addition of even 10% NaBH_4_ in the synthesis of bionanohybrids considerably improves their metallic activity in both oxidation and reduction reactions.

Neither the species nor the copper content differ too much between **Cu-PHOS-0% R** and **Cu-PHOS-10% R**. The most significative difference between them can be seen in the formation of different nanostructures in **Cu-PHOS-10% R**. In addition, the TEM images of the other bionanohybrids with a higher percentage of the reducing agent and a better efficiency in pAP and pNP reactions also show the presence of nanostructures. Other studies have previously demonstrated^18,28^ that the presence of these structures gives to the bionanohybrids a higher efficiency and enhanced properties compared with the nanomaterials with no remarkable nanostructures, as **Cu-PHOS-0% R**.

### 3.3 Evaluation of Antimicrobial Activity of Copper Bionanohybrids

The antimicrobial activity of copper bionanohybrids was tested against Gram-negative and Gram-positive bacteria through two different methods.

**Figure 6:**
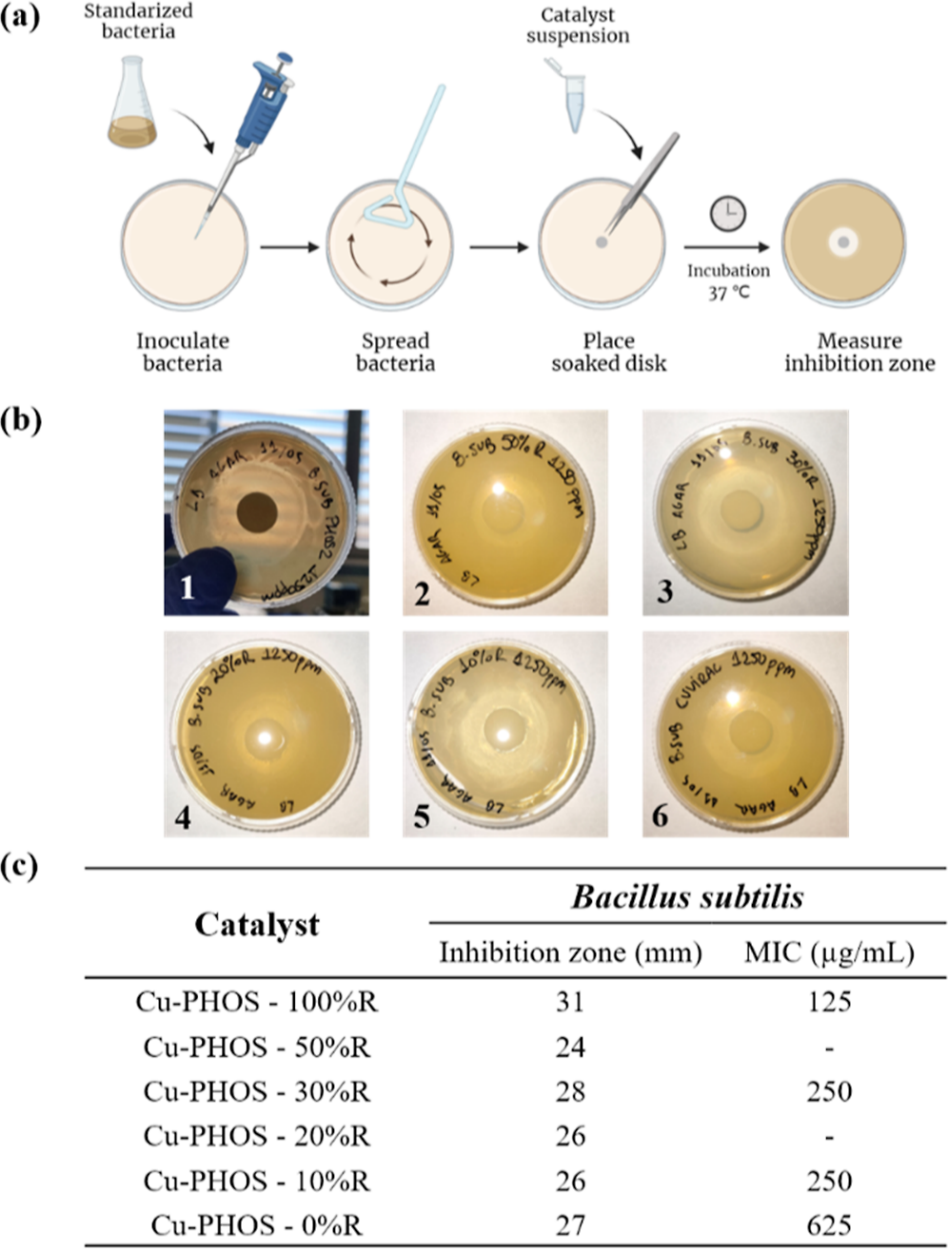
Disk diffusion assay: (a) scheme of the method. Created with BioRender.com; (b) LB-agar plates after *B. subtilis* incubation with the bionanohybrids (1250 ppm). (1) **Cu-PHOS-100% R**, (2) **Cu-PHOS-50% R**, (3) **Cu-PHOS-30% R**, (4) **Cu-PHOS-20% R**, (5) **Cu-PHOS-10% R**, and (6) **Cu-PHOS-0% R**; (c) table of results of inhibition zones measured for a bionanohybrid concentration of 1250 ppm and MIC values.

First, the antimicrobial efficacy against *B. subtilis* was tested with the disk diffusion method (Figure 6a,b). The inhibition zones were measured after an incubation time of 2 days with a concentration of the bionanohybrids of 1250 μg/mL. The inhibition zones obtained were from 24 to 31 mm (Figure 6c). Specifically, these data corresponded to **Cu-PHOS-50% R** and **Cu-PHOS-100% R** bionanohybrids, respectively. The inhibition zones measured for **Cu-PHOS-30% R**, **Cu-PHOS-20% R**, **Cu-PHOS-10% R**, and **Cu-PHOS-0% R** were very similar, from 26 to 28 mm.

After that, the best bionanohybrids (**Cu-PHOS-100% R**, **Cu-PHOS-30% R**, **Cu-PHOS-10% R**, and **Cu-PHOS-0% R**) were selected to determine the MIC. Five decreasing concentrations from 1250 to 125 μg/mL of those bionanohybrids were tested, and the results can be found in Figure 6c.

Both **Cu-PHOS-30% R** and **Cu-PHOS-10% R** showed the same MIC, 250 μg/mL. **Cu-PHOS-0% R** presented the higher MIC, 625 μg/mL. The bionanohybrid with the best results was **Cu-PHOS-100% R**, with a 125 μg/mL MIC. The difference in the copper species was again highlighted, being the hybrid with Cu(I) and Cu(0) species, **Cu-PHOS-100% R**, the one with the greatest antibacterial effect against *B. subtilis*.

However, the differences in the antibacterial effect of **Cu-PHOS-30% R**, **Cu-PHOS-20% R**, and **Cu-PHOS-10% R** was minimum. In addition, the variations in the inhibition zone and the MIC between them were not significant. Nevertheless, the fact of not adding any reducing agent generated a less efficient bionanohybrid, as can be seen in MIC data for **Cu-PHOS-0% R**.

Therefore, in order to get a more sustainable bionanohybrid but sufficiently efficient, we could decrease the amount of the reducing agent by up to 10% compared to the original one, even though the antimicrobial efficiency would be slightly reduced.

**Figure 7:**
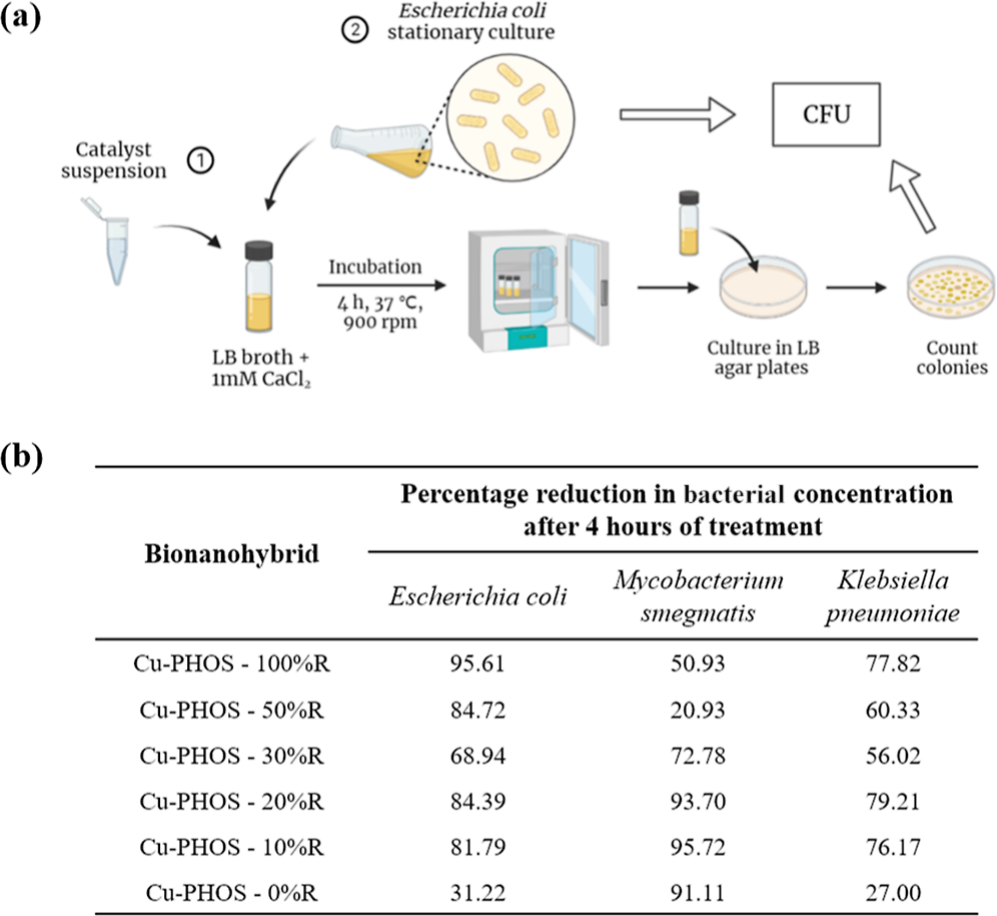
Antimicrobial assay of bionanohybrids by counting the CFU: (a) scheme of the method. Created with BioRender.com; (b) results table of percentage reduction in bacterial concentration (measured viable bacteria as CFU count per mL in the presence of the bionanohybrid relative to the control in the absence of the bionanohybrid) after a 4 h incubation with a concentration 1250 ppm of each bionanohybrid.

Subsequently, antimicrobial analyses were performed with an *E. coli* strain by counting colony-forming units (CFU) after incubating a stationary culture of bacteria for 4 h with each bionanohybrid in a concentration of 1250 ppm (Figures 7a, S3). This methodology has been based on previously developed procedures.^29,30^

As is shown in Figure 7b, the bionanohybrid with the best antimicrobial capacity against *E. coli* was **Cu-PHOS-100% R**, with a percentage of bacteria reduction of 96%. It was followed by **Cu-PHOS-50% R** and **Cu-PHOS-20% R** and **Cu-PHOS-10% R**, with more than 81% of efficacy. **Cu-PHOS-30% R** and **Cu-PHOS-0% R** were the less efficient with a 69 and 31% of efficacy, respectively.

The antimicrobial effect of the bionanohybrids was tested against two extra bacterial strains, *M. smegmatis* and *K. pneumoniae*, following the same procedure performed previously with *E. coli*. It was obtained that **Cu-PHOS-0% R** had the best antimicrobial effect against *M. smegmatis*, with 96% concentration reduction in 4 h. It was followed by **Cu-PHOS-10% R** and **Cu-PHOS-100% R**, with efficiencies of 94 and 91%, respectively. Reduction percentages were slightly lower for *K. pneumoniae*, with a maximum of 79% for **Cu-PHOS-10% R**.

Experimental results indicate that bionanohybrids have antimicrobial effects in different intensity levels depending on the bacterial strain and the synthesis conditions of each one of them. In general, against Gram-positive bacteria (*B. subtilis* and *M. smegmatis*), both the most reduced and the least reduced have good antimicrobial effect, with no clear distinction. Thus, the best bionanohybrid against *B. subtilis* was **Cu-PHOS-100% R**, while against *M. smegmatis* was **Cu-PHOS-10% R**. In both cases, **Cu-PHOS-0% R** was also very efficient, being among the best ones.

However, against Gram-negative bacteria (*E. coli*, *K. pneumoniae*), reduced bionanohybrids seem to give better results, regardless of the amount of the reducing agent. It should be stressed that for the two strains tested, the unreduced bionanohybrid, **Cu-PHOS-0% R**, gave the lowest results. In this case, the difference in the efficiency of **Cu-PHOS-0% R** may be due to the lack of nanostructures, which are present in the reduced bionanohybrids, since the predominant specie (Cu^2+^) is the same as in other bionanohybrids. Nevertheless, it seems that this factor, so significant against Gram-negative bacteria, would not be of importance against Gram-positive bacteria, where **Cu-PHOS-0% R** is efficient and where the species seems to be the key factor. On the other hand, data obtained with Gram-negative bacteria are in agreement with the results obtained in pAP oxidation assays, so that pAP assay may be useful as a first approximation for the evaluation of antimicrobial activity in these strains.

The bionanohybrid with the highest amount of reducing agent, **Cu-PHOS-100% R** (with the presence of Cu(I) and Cu(0) species, an average particle size of 11 nm and large nanostructures), stands out for the good results achieved against different strains. This indicates that Cu(I) and Cu(0) have an antimicrobial powder much greater than other copper species.

To finally verify this parameter, the disk diffusion assay was tested with **Cu-BIC-100% R**, in which Cu(0) species predominate, as previously mentioned. The inhibition zone measured for a bionanohybrid concentration of 1250 ppm was 30 mm. Comparing **Cu-BIC-100% R** with **Cu-PHOS-100% R**, where the predominant copper species are Cu(0) and Cu(I), respectively, it can be seen that the antimicrobial activity is approximately the same. Thus, the results showed that the efficiency between Cu(0) and Cu(I) against bacteria is almost identical.

By contrast, if we compare the antimicrobial efficiency of the copper bionanohybrids developed in this study with CuNPs structured in chitosan^31^ or leaf extracts,^32^ we find better results for our enzyme-NPs, translated in major inhibition zones and lowest MIC values against *B. subtilis*. This suggests that the bionanohybrids designed give stability and efficiency to the CuNPs, resulting in an optimal and sustainable synthesis method for new nanobiomaterials with antimicrobial properties.

## 4 Conclusions

In this study, we have presented a simple but versatile synthesis protocol to prepare CuNP hybrids with antimicrobial activity in mild conditions at room temperature. The method is based on the use of an enzyme as a scaffold that induces the formation of NPs in the protein network and generates different metallic species, NPs sizes, and morphologies depending on the percentage of the reducing agent added.

The results obtained have shown the high antimicrobial efficiency of some of the designed bionanohybrids against Gram-positive and Gram-negative bacteria. The bionanohybrid with the best performance in the disk diffusion assay against *B. subtilis* was **Cu-PHOS-100% R**, showing an inhibition zone of 31 mm and a MIC of 125 μg/mL. In addition, this bionanohybrid showed high percentages of *E. coli* and *K. pneumoniae* reduction in the CFU assay. Other bionanohybrids (with a lower reducing agent in the synthesis) showed the best antimicrobial behaviors against Gram-positive bacteria, reaching reduction percentages above 90% for *M. smegmatis* and inhibition zones against *B. subtilis* above 25 mm. Moreover, the results suggest that the efficiencies vary according to the synthesis method, being more effective against Gram-negative bacteria, the bionanohybrids in which more reducing agents have been used, which is explained by the different nanostructures formed in the material.

Thus, the antimicrobial activity found in these new bionanohybrids opens the possibility of using them as novel antimicrobial agents, which can be applied as disinfecting agents in places where antibiotic-resistant bacteria are a problem or be quite interesting for microbiologically influenced corrosion (MIC) mitigation.

## Supporting Information Available

Explanation of the mechanism of NP formation, general scheme of the synthesis of copper nanohybrids, viable bacterial concentration, measured as colony count per mL (CFU/mL) in the absence of the bionanohybrid and in the presence of the bionanohybrid, and table collecting their different characteristics (PDF)

## Abbreviations

CAL-B: lipase B from *C. antarctica*
CFU: colony-forming units
CuNPs: copper nanoparticles
LB: Luria–Bertani
MIC: minimum inhibitory concentration
NPs: nanoparticles
pAP: *p*-aminophenol
pNP: *p*-nitrophenol
ROS: reactive oxygen species
SEM: scanning electron microscopy
TEM: transmission electron microscopy
XRD: X-ray diffraction
