# Supplementary material for: Design and Synthesis of Copper Nanobiomaterials with Antimicrobial Properties
Source: ACS Bio Med Chem Au. 2023 Apr 11;3(4):349–58. doi: 10.1021/acsbiomedchemau.2c00089 (PMC10436259; doi:10.1021/acsbiomedchemau.2c00089)
Supplement: Supplementary file 1 — bg2c00089_si_001.pdf [file bg2c00089_si_001.pdf]

Supporting Information

Design and synthesis of copper nanobiomaterials with antimicrobial properties

Clara Ortega-Nieto[1], Noelia Losada-Garcia[1], Benevides C. Pessela[2], Pilar Domingo-Calap[3], Jose M. Palomo[1,*]

[1] Instituto de Catalisis y Petroleoquimica (ICP), CSIC, Marie Curie 2, 28049, Madrid, Spain

[2] Institute of Food Science Research (CIAL, CSIC-UAM), Nicolás Cabrera, 9, Cantoblanco, 28049, Madrid, Spain

[3] Institute for Integrative Systems Biology (I[2]SysBio), Universitat de València-CSIC, 46980, Paterna, Spain

* Correspondence: josempalomo@icp.csic.es; Tel.: +34-9158-5476-8

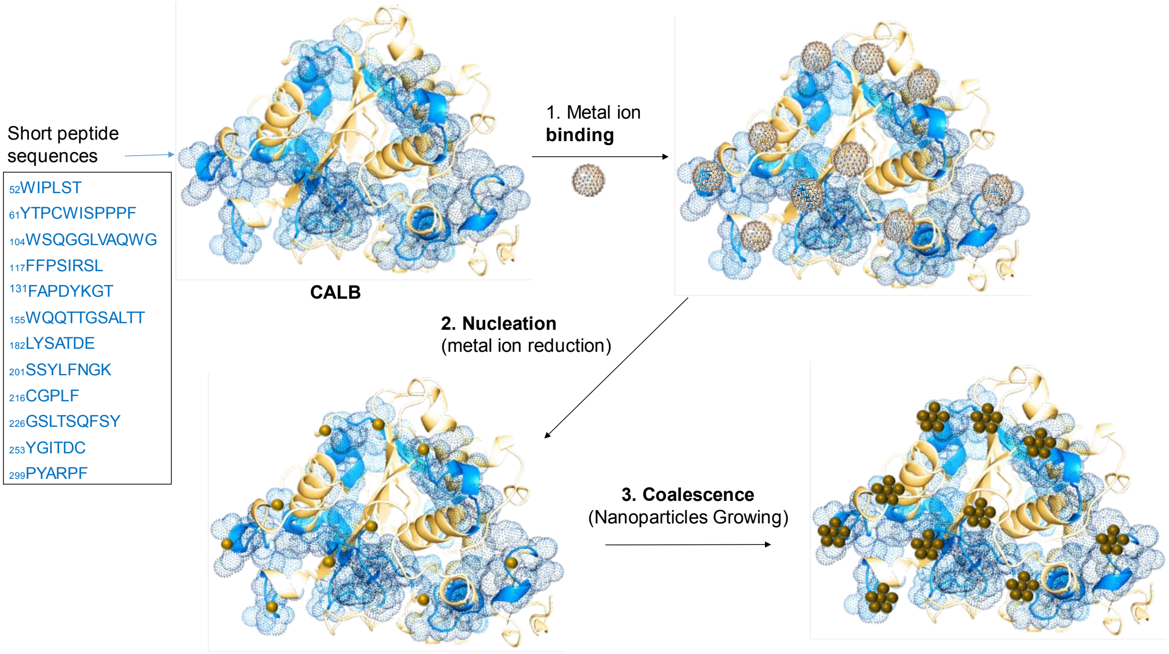

Figure S1. Proposed mechanism of metal nanoparticles formation using CALB as scaffold. Nucleation is performed in the case of copper using $NaBH_4$ as reducing agent.

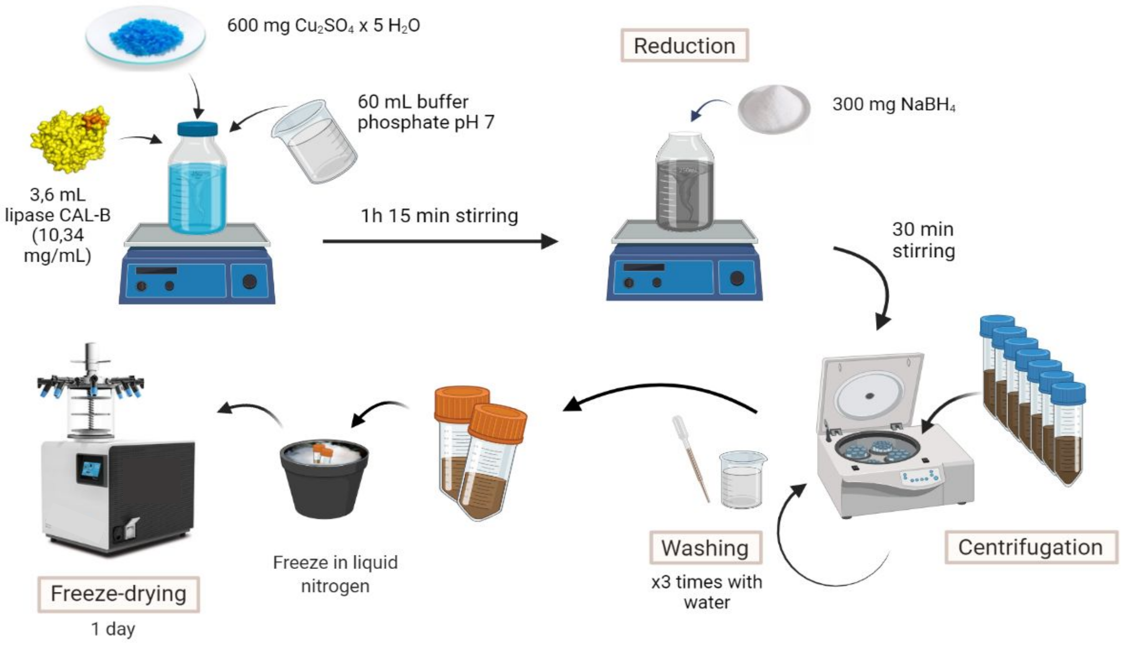

Figure S2. Scheme of bionanohybrids synthetic protocol.

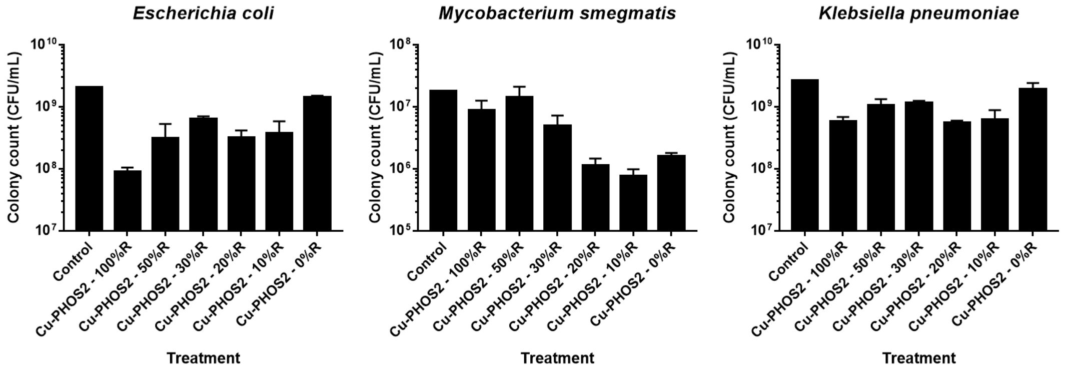

Figure S3. Viable bacterial concentration, measured as colony count per mL (CFU/mL) in absence of the bionanohybrid (control) and in the presence of the bionanohybrid (with a concentration 1250 ppm of each bionanohybrid) after a 4h incubation.

Table S1. Different properties of the synthesized bionanohybrids

Name	% of reducing agent added	Species detected in XRD	Average particle size (nm)	Copper content (%)
**Cu-PHOS-100%R**	100%	Cu(0) and Cu_2O	11.4 ± 1.8	45
**Cu-PHOS-50%R**	50%	Cu_2O and $Cu_3(PO_4)_2$	4.9 ± 1.2	37
**Cu-PHOS-30%R**	30%	$Cu_3(PO_4)_2$	5.9 ± 1.0	55
**Cu-PHOS-20%R**	20%	$Cu_3(PO_4)_2$	5.6 ± 1.1	29
**Cu-PHOS-10%R**	10%	$Cu_3(PO_4)_2$	5.6 ± 1.0	27
**Cu-PHOS-0%R**	0%	$Cu_3(PO_4)_2$	3.9 ± 0.5	32
